# Practice and Impact of Multidisciplinary Tumor Boards on Patient Management: A Prospective Study

**DOI:** 10.1200/JGO.2016.004960

**Published:** 2016-08-10

**Authors:** Raghid N. Charara, Firas Y. Kreidieh, Rania A. Farhat, Karine A. Al-Feghali, Katia E. Khoury, Ali Haydar, Lara Nassar, Ghina Berjawi, Ali Shamseddine, Nagi S. El Saghir

**Affiliations:** **Firas Y. Kreidieh**, **Karine A**. **Al-Feghali**, **Ali Haydar**, **Lara Nassar**, **Ghina Berjawi**, **Ali Shamseddine**, and **Nagi S. El Saghir**, American University of Beirut Medical Center, Beirut, Lebanon; **Raghid N. Charara**, Institute for Health Metrics and Evaluation, Seattle, WA; **Rania A. Farhat**, St Louis University Hospital, St Louis, MO; and **Katia E. Khoury**, University Hospitals Case Medical Center, Cleveland, OH.

## Abstract

**Purpose:**

Multidisciplinary tumor boards (MTBs) have become commonplace. The use, attendance, and function of MTBs need continued assessment and improvement.

**Methods:**

We prospectively recorded and assessed all cases presented at MTBs between October 2013 and December 2014. Data were collected before and during each MTB. Data were analyzed using SPSS for Windows version 23 (SPSS, Chicago, IL).

**Results:**

Five hundred three cases were presented: 234 cases (46%) at GI cancer MTBs, 149 cases (29.6%) at breast cancer MTBs, 69 cases (13.7%) at thoracic/head and neck cancer MTBs, and 51 cases (10.7%) at neuro-oncology MTBs. A total of 86.7% of MTB cases were presented to make plans for management. Plans for upfront management were made in 67% of the breast cancer cases, 63% of GI cases, 59% of thoracic/head and neck cases, and 49% of neuro-oncology cases. Three hundred ninety-four cases (78.3%) were presented by medical oncologists, whereas only 74 cases (14.7%) were presented by surgeons, and 10 cases (2%) were presented by radiation oncologists. The majority of MTBs, with the exception of the neurosurgery MTBs, were led by medical oncologists. Surgeons presented the least number of cases but attended the most, and their contributions to discussions and decision making were essential.

**Conclusion:**

MTBs enhance the multidisciplinary management of patients with cancer. Upfront multidisciplinary decision making should be considered as an indicator of benefit from MTBs, in addition to changes in management plans made at MTBs. Increasing the contributions of surgeons to MTBs should include bringing more of their own cases for discussion.

## INTRODUCTION

Cancer care is becoming increasingly complex, and input from various specialists and team members has become pivotal for proper management. Multidisciplinary management can be performed either by specialized units, such as breast units, as is most frequently practiced in Europe, or by conducting multidisciplinary tumor boards (MTBs), a practice that is most frequently in the United States of America and that is becoming popular worldwide.^1-3^ MTBs are forums for multidisciplinary management of patients with cancer; a group of physicians from different specialties (medical oncologists, radiation oncologists, radiologists, pathologists, surgeons and others) convene to discuss the cases of patients with cancer.^1,4^ Clinical cases, including laboratory, imaging, and pathology findings are presented. Findings are debated and discussed, after which group recommendations are made. Depending on the MTB structure, time management, and the expertise of the leadership, discussions may lead to more accurate diagnoses and evidence-based up-to-date management plans.^1,2,5^ A recent survey of ASCO international membership showed that MTBs are used by 85% of respondents in their clinical practice.^2^

A few single institution studies have reported changes in diagnosis and/or treatment plans in 20% to 50% of cases presented at MTBs, particularly cases of breast cancer.^6-10^A multi-institutional survey by Keating et al^11^ found little association between Veterans Administration Hospitals’ MTBs and patient quality of life or survival. An accompanying editorial^12^ and correspondence replies^13-16^ stressed the importance of improving the structure and quality of MTB discussions,^4,17,18^ the importance of MTBs in the management of patients with breast cancer,^14,19,20^ and the value of mini–tumor boards in areas in which not all specialists are available.^21,22^

To assess the use and efficiency of MTBs in a prospective manner, we designed this study to look at the process of case presentations, plans for therapy, changes in diagnoses and treatment plans for various types of cancer, and help in patient management at weekly MTBs meetings. We discuss our findings and lessons learned about the assessment and advancement of multidisciplinary patient care for patients with cancer.

## METHODS

The study involved assessment of all cases presented at the various tumor boards at the American University of Beirut Medical Center (AUBMC); it was approved by the institutional review board. We prospectively recorded all cases presented at AUBMC tumor boards for 14 months, between October 2013 and December 2014. Data were collected before and during each MTB. To avoid any possible bias, data were collected by the research team and not the attending physicians. Data were analyzed by SPSS for Windows version 23 (SPSS, Chicago, IL). Patient confidentiality was maintained during the MTBs and throughout the study; during data entry and analysis of results, each case was coded by a unique identification number, and attending physician names were also coded numerically.

Data collected during MTBs included patients’ demographics, initial findings by history, physical examination results, radiology reports, pathology reports, relevant laboratory values, the primary physician’s diagnosis in terms of type of cancer and staging, and the primary physician’s initial plan of management. Data were completed as needed from the patients’ hospital records. Each case was then assessed for changes in diagnosis (clinical, pathologic, laboratory, or radiologic), a difference in staging, or a difference in management plan before and after tumor board discussions. The form used for data collection is shown in the Data Supplement. The primary end point was evaluation of change in plans resulting from tumor board discussions.

The structure and leadership of the MTBs was noted from our attendance and continuing medical education credits sheets. The attendance of each person was noted for each type of MTB conducted, and the probability that he/she would attend tumor boards of that particular type was calculated.^23^ Four types of MTBs were conducted on the basis of the cases presented in each: GI cases, breast cancer cases, neuro-oncology cases, and head and neck cases. For each attendee, we calculated the ratio of MTBs attended to the total number of MTBs conducted for every type of MTB. This represents the probability that each attendee would attend a MTB of that particular type. Each attendee was then assigned to one of five specialty groups: medical oncologists, radiation oncologists, surgeons, radiologists, and pathologists. The probabilities of all attendees belonging to a particular specialty group were averaged. This represents the probability that an attendee belonging to a particular specialty group would attend a MTB of a particular type. In addition, to obtain the overall probability for each specialty group of attendees, we combined the attendance sheets of all attendees from all the MTBs and calculated the probabilities for all attendees and for each specialty group of attendees as indicated previously. We included attending physicians only and excluded residents and fellows-in-training.

## RESULTS

Five hundred three cases were presented between October 2013 and December 2014 from general and subspecialty cancer-specific tumor boards (neuro-oncology, GI, breast, thoracic/head and neck tumor boards.

**Fig 1:**
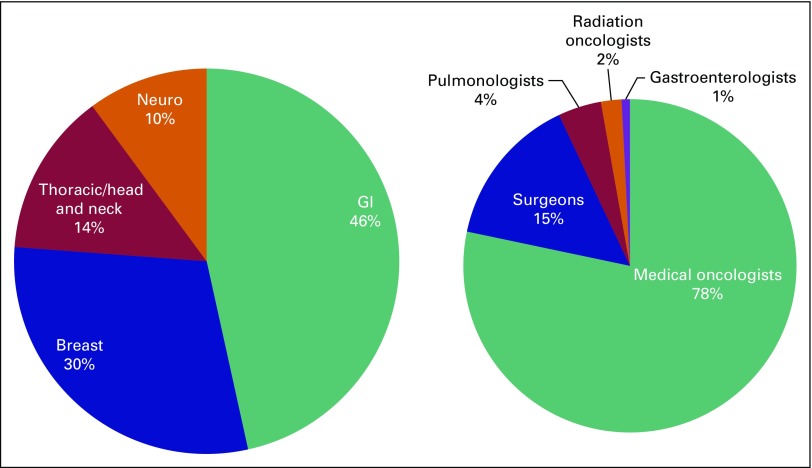
Distribution of cases presented at multidisciplinary tumor boards by type of cancer and specialty of presenter. Neuro, neuro-oncology.

Of the 503 cases presented at the tumor boards, 234 cases were GI cancer (46.5% of total cases), 149 cases were breast cancer (29.6%), 69 (13.7%) were thoracic/head and neck cancer, and 51 (10.7%) were neuro-oncology cases (Fig 1).

### Reasons for Presentations for All Cases

Of the 503 cases, only 13.3% had a plan of management before presentation at tumor boards, and 86.7% of tumor board cases were presented by the treating physician to make plans for management (Table 1). When we looked at changes in diagnostic plans and therapeutic plans separately, physicians sought a diagnostic plan in 35.6% of cases and sought a therapeutic plan in 75% of the cases. Eighty-six cases (17.1%) were presented to finalize pathology and/or radiology results. (Numbers add up to more than 100% because of overlap between diagnosis and treatment plans). Sixty-seven cases (13.3%) were presented for educational value and follow-up information (Table 1).

**Table 1:**
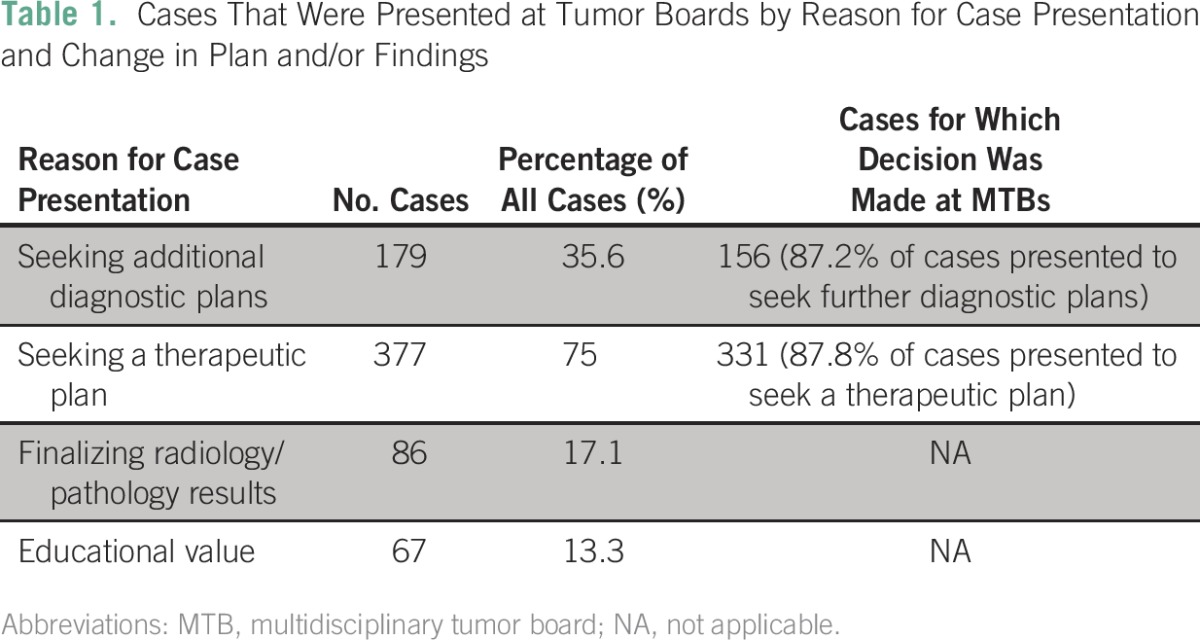
Cases That Were Presented at Tumor Boards by Reason for Case Presentation and Change in Plan and/or Findings

### Changes and Decision Making at MTBs

Of the 377 cases presented for therapeutic planning, the majority, 331 (87.8% of 377 cases), had therapeutic plans made at the MTB itself (Table 1). Of the 377 cases, 47 were presented by physicians who had prior plans for treatment. No changes were made in 39 of them (84.8% of 46 cases); changes were made in only seven (15.2%).

Of the 179 cases presented for diagnostic planning, the majority, 156 (87.2% of 156 cases), had diagnostic plans made at the MTB (Table 1). Of the 179 cases, 23 were presented by physicians who had prior diagnostic planning. No changes were made in 19 of them (82.6% of 23 cases); changes were made in only four (17.4%).

Of the 67 cases with a plan made before MTB case presentation (Table 1), four (6%) had a change in therapeutic plan, four (6%) had a change in diagnostic plan, and one (1.5%) had a change in diagnosis (Table 2). Examples of management plan options include giving chemotherapy, giving hormonal therapy, performing surgery, and giving radiation therapy. Of the 67 cases whose plan was made before MTB discussion, physicians had a plan to give chemotherapy or hormonal therapy in 19 cases (28.36%), had a plan to perform surgery in 17 cases (25.37%), and had a plan to give radiation therapy to patients in six cases (8.96%). (Numbers do not add to 67 because there were other plans, such as embolization, for example, that were also present for a minority of cases.) In addition, 12 cases had a change in radiology findings (Table 2), and six cases had a change in pathology findings (Table 2).

**Table 2:**
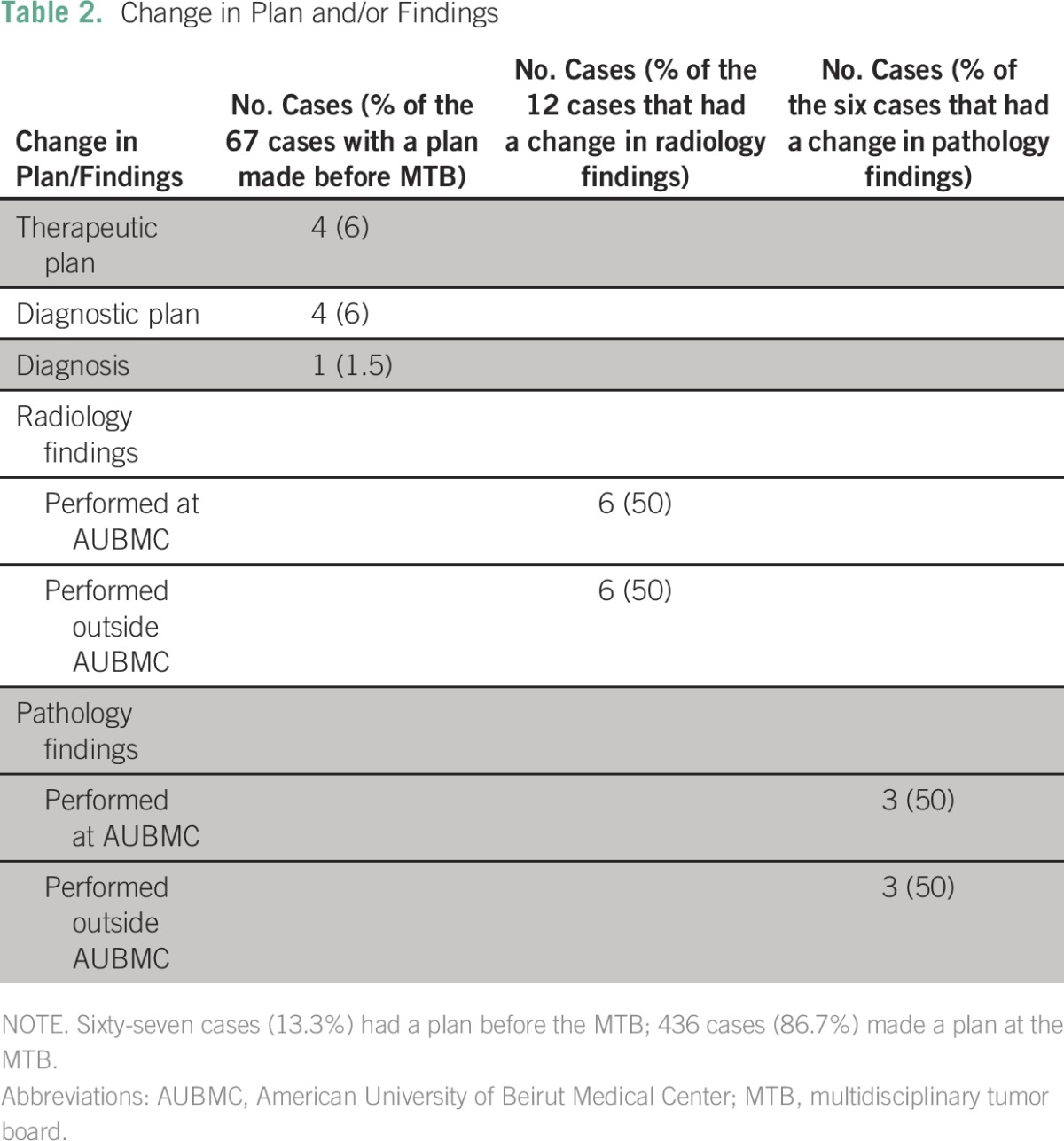
Change in Plan and/or Findings

When we looked at cases by subspecialty, physicians sought plans for management in 91.3% of GI cases presented at MTBs, in 83.2% of breast cases, in 87% of thoracic/head and neck cases, and in 58.8% of neuro-oncology cases. Table 3 summarizes the reasons for case presentation and plans made at MTBs.

**Table 3:**
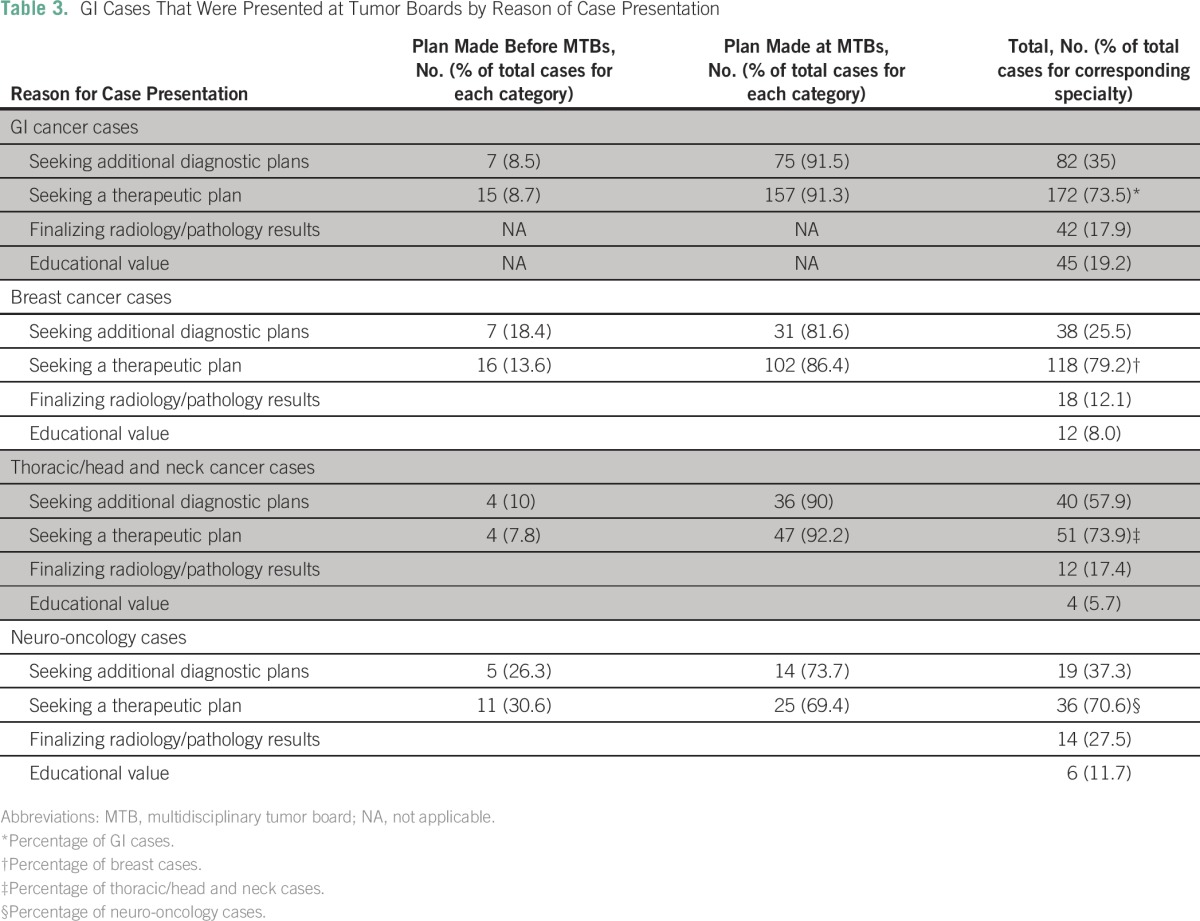
GI Cases That Were Presented at Tumor Boards by Reason of Case Presentation

### MTB Leadership and Attendance

We assessed the attendance of physicians by their specialty. All MTBs, with the exception of neuro-oncology MTBs, were chaired by a medical oncologist. All MTBs had at least one physician from each specialty. To assess the average attendance from each particular physician and each specialty throughout the study period, we used probability.^23^ Physicians attend according to their schedule, the travel involved, their interest, and the particular cases presented. Medical oncologists had the highest probability of attending a tumor board, with a mean probability of 46.37%. They were followed by radiation oncologists, who had a probability of 39.66%. Radiologists and surgeons had a lower probability of attending a MTB, with respective mean probabilities of 22.77% and 19.71%. As for pathologists, the probability of attending a tumor board was 13.89%. MTBs conducted at AUBMC are equipped with screen projectors, access to an online IMPAX radiology system installed at AUBMC by AGFA Healthcare for digital software image viewing reporting, pathology slides, a microscope, and a projector. This is a favorable setting to encourage radiologists, pathologists, and surgeons to attend and contribute to management decisions. Attendance by surgeons and radiologists (especially interventional radiologists) could have been affected by surgeries and breast biopsies, respectively. Indeed, on reviewing attendance sheets, we noted many absences for surgeons and radiologists because of unscheduled operations and breast biopsies, respectively.

For each attendee, we calculated the ratio of MTBs attended to the total number of all MTBs conducted for every type of MTB. This represents the probability that each attendee would attend a MTB of that particular type. Each attendee was then assigned to one of five specialty groups: medical oncologists, radiation oncologists, surgeons, radiologists, and pathologists. The probabilities of all attendees belonging to a particular specialty group were averaged. This represents the probability that an attendee of a particular specialty group would attend a tumor board of a particular type. In addition, to obtain the overall probability for each specialty group of attendees, we combined the attendance sheets of all attendees from all the MTBs and calculated the probabilities for all attendees and for each specialty group of attendees, as indicated previously. We included attending physicians only and excluded residents and fellows-in-training.

When we stratified by type of MTB, we found that surgeons had probabilities that reached 90.00% and 69.82% of attending neuro-oncology and GI tumor boards, respectively. This was lower for breast and head and neck MTBs, with probabilities reaching 25.29% and 20.37%, respectively. Radiation oncologists had a probability of attending a GI MTB that reached 87.80%. This probability decreased to 45.83%, 33.95%, and 20.37% for head and neck, neuro-oncology, and breast tumor boards, respectively. Medical oncologists had a probability of attending tumor boards that was highest for GI MTBs, at 61.89%. The probability was similar for each of head and neck, neuro-oncology, and breast MTBs, with probabilities of 29.16%, 37.77%, and 37.59%, respectively. Radiologists had the highest probability of attending GI and breast tumor boards, with probabilities of 63.41% and 44.52%, respectively. This decreased to 37.03% for head and neck MTBs and to 13.66% for neuro-oncology MTBs. Pathologists’ probability of attending GI MTBs seemed to be similar to that of radiologists, at 60.97%. This probability decreased for head and neck, neuro-oncology, and breast MTBs with probabilities of 16.65%, 27.77%, and 29.07%, respectively.

### Case Presentations and Discussions

Of the 503 cases, 394 cases (78.3%) were presented by medical oncologists, 74 cases (14.7%) were presented by surgeons, 21 cases (4.2%) were presented by pulmonologists, 10 cases (2%) were presented by radiation oncologists, and four cases (0.8%) were presented by gastroenterologists (Fig 1). All physicians participated in case discussions and in the making of management plans. Although the majority of cases were presented by medical oncologists, surgeons and radiation oncologists contributed to discussions and management plans.

## DISCUSSION

Although our study was designed with the primary end point of assessing changes of plans of patient management at MTBs, our main finding was that the majority of multidisciplinary plans of management are made at the MTBs. This study shows that MTBs are used as a forum for group consultations and multidisciplinary patient management. We view this as a positive trend toward better and multidisciplinary care of patients with cancer. For example, plans for new treatment were made in 67% of the breast cancer cases, 63% of the GI cases, 59% of the thoracic/head and neck cases, and 49% of the neuro-oncology cases.

We noted from our study that more multidisciplinary plans were made at GI MTBs. This might be explained by current trends for recommendations for multidisciplinary management, including radiation therapy and surgery, for almost all cases, for both early-stage and metastatic colorectal cancer.^10^

When physicians had already made a plan of management before the MTB, changes in diagnosis were seen in 16 cases only, and changes in therapeutic plan were seen in seven cases only. Cases that had a change in diagnosis were divided among the specialties as follows: 2.6% for GI cases, 2.7% for breast cases, 5.8% for thoracic/head and neck cases, and 3.9% for neuro-oncology cases.

We also noted that medical oncologists were more likely to bring cases to the MTBs than were surgeons or radiation oncologists. A recent publication from the Cancer Care Outcomes Research and Surveillance Consortium (CanCORS) prospective observational survey assessing care patterns of patients with lung and colorectal cancer showed that surgeons are less likely to attend weekly MTBs than are medical oncologists and radiation oncologists. The CanCORS study showed that frequent physician MTB engagement was associated with more clinical trial participation and with high rates of curative-intent surgery for early-stage non–small-cell lung cancer, but not with overall survival. The authors also mentioned that in exploratory subgroup analysis, frequent tumor board participation was associated with improved survival among patients with extensive small-cell lung cancer and stage IV colorectal cancer, and they noted that MTBs may be most beneficial for complex cases and unusual clinical scenarios.^10^

In a previous publication, we noted that MTBs are also beneficial in remote areas or in low-resource settings, where only a small group of specialists is available, in which case a mini–tumor board may be held with whomever is available to make better management decisions and plans.^5,21^ Videoconferencing can be useful in such circumstances. Current advances in technology allow videoconferencing and virtual tumor boards through Web conferencing platforms to be made available, allowing all physicians, including those practicing in remote areas, to make multidisciplinary management plans for their patients.^23,24^ At our institution, we hold separate monthly educational MTB videoconferences with Memorial Sloan Kettering Cancer Center and with MD Anderson Cancer Center.^25,26^

Improvement in the efficiency of MTBs is needed, and suggestions were published in our recently published survey of the international membership of ASCO. More effective moderators of discussions, better time management at meetings, better criteria for the selection of cases, and the provision of written summaries of cases to attendees can better reduce the time and resources needed for MTBs.

Although medical oncologists presented the majority of cases at our institution, surgeons were essential in discussions and final management plans. This may vary from institutions where surgeons or radiation oncologists lead. All members of the multidisciplinary management team are important contributors to the success of MTBs by virtue of case preparation, presentation, discussion, and the making of management plans. Radiologists and surgeons had lower probabilities of attending a MTB, with respective mean probabilities of 22.77% and 19.71%. As for pathologists, the probability of attending a tumor board was 13.89%. This may be because of other obligations, including scheduled operations and procedures for surgeons and radiologists, respectively. As much as participation in discussions and decision making is important, presentation of cases would expand multidisciplinary care to all patients with cancer at each institution.

These results from our institution show that the use of MTBs enhances the implementation of multidisciplinary management of patients with cancer and should be helpful to health care providers worldwide, including those practicing in remote areas and in low- and middle-income countries.

In conclusion, multidisciplinary management of patients with cancer is recommended for the modern practice of oncology. Change in management plans is considered an indicator of the benefit of MTBs. Our study shows that tumor boards are frequently used to make initial multidisciplinary plans for patient management. In addition to considering change in management plans as an indicator of the benefit of MTBs, we suggest that making upfront multidisciplinary plans for patient management be considered an additional component of indicators of the benefit of MTBs. Surgeons presented the lowest number of cases at our institution MTBs, but they attended and participated more in discussions and decision making. Enhancement of multidisciplinary team work and further research, in addition to the use of virtual tumor boards and videoconferencing, are warranted. The effects on management, outcome, and survival should be assessed at various centers and for different cancers.
